# Exploring hospitals’ functional preparedness effective factors in response to disasters: a qualitative study in a lower middle-income country

**DOI:** 10.1186/s12913-024-10630-y

**Published:** 2024-02-13

**Authors:** Behrouz Samei, Javad Babaie, Jafar Sadegh Tabrizi, Homayoun Sadeghi-bazargani, Saber Azami Aghdash, Naser Derakhshani, Ramin Rezapour

**Affiliations:** 1 Tabriz Health Services Management Research Center, Tabriz University of Medical Sciences, Tabriz, Iran; 2 Department of Health Policy & Management, Tabriz Health Services Management Research Centre, School of Management & Medical Informatics, Tabriz University of Medical Sciences, Tabriz, Iran; 3 Road Traffic Injury Research Center, Tabriz University of Medical Sciences, Tabriz, Iran; 4 Medical Philosophy and History Research Center, Tabriz University of Medical Sciences, Tabriz, Iran; 5 Health Management and Economics Research Center, Health Management Research Institute, Iran University of Medical Sciences, Tehran, Iran

**Keywords:** Functional preparedness, Hospital, Disaster, Response, Disaster Medicine

## Abstract

**Background:**

Medical services are among the most urgent needs of the disaster-affected population. Consequently, hospital preparedness -as the main health services provider- is one of the vital factors in effective response to disasters. The present study aims to explore the perspectives of study participants about the influential factors of hospital functional preparedness in a lower middle-income country.

**Methods:**

In this qualitative study, data were collected through 17 semi-structured interviews with disaster management authorities selected by purposive sampling. Content-Analysis was used to analyze the data.

**Results:**

138 codes were developed and categorized into ten categories and 34 subcategories. The main categories were: 1- leadership, command, and coordination (4 subcategories); 2- risk assessment (3 subcategories); 3- legislating and developing protocols, guidelines, and programs (3 subcategories); 4- estimating and storing the necessary supplies and equipment (3 subcategories); 5- human resource management (4 subcategories); 6- education, training, and development of staff (6 subcategories); 7- vital routes and facilities (3 subcategories); 8- communication (3 subcategories); 9- security, safety and locating of safe zones (3 subcategories); 10- underlying disaster risk factors (2 subcategories).

**Conclusion:**

According to the participants of this study, ten categories of factors can affect hospitals’ functional preparedness; hospital managers and decision-makers can consider these factors to ensure the proper provision of medical services during disasters.

**Supplementary Information:**

The online version contains supplementary material available at 10.1186/s12913-024-10630-y.

## Background

Disasters are serious disruptive events with widespread consequences such as death, injury, and financial loss that exceed the capacity of affected communities to adapt using their local available resources [1, 2]. The history of humanity reveals a rise in the frequency and severity of disasters. Almost less developed countries have spent substantial resources to respond [3, 4]. Disasters are common phenomena in the world. For instance, in 2021, all around the world, 432 natural hazards induced disasters were reported that led to the death of 10,492, affected more than 101 million, and resulted in 252 billion US$ in damages [5]. They affect all economic, political, and cultural issues, damage infrastructure, and overwhelm health systems with a large number of victims for an extended period [6, 7]. Many governments allocate roughly 3% of their annual gross domestic product to disaster damage compensation [8].

Iran is one of the disaster-prone nations [9]. It is located on the most significant seismic belt in the world (The alpine-Himalayan seismic belt) [9]. More than 3,500 rivers in the country have the potential to cause floods; Different regions, diverse climates, and climate change have also increased risks. Historical earthquakes in Bam, Rudbar, Tabas, Gilan, and Zanjan, two recent tragic earthquakes in Kermanshah [10] and East Azerbaijan [11], and the Golestan province flood are examples of these devastating disasters [12].

Regarding disaster vulnerability, Iran ranks tenth in the world and fourth in Asia; Iran accounts for 6% of all disasters worldwide [7, 13]. Between1970-2010, 10,105 natural hazards occurred in Iran. In these forty years (1970–2010), more than 153,000 people lost their lives due to natural hazards, more than 42 million have been affected, and nearly 154,000 have been injured [14].

Disasters result in infrastructure disruption and increased human needs [15]. The most important of which is health services [16]. According to the evidence, the effective response of health systems can be an influential factor in reducing disaster-related fatalities [17–20]. Among healthcare providers, hospitals, as the first place to which people refer, must be prepared to deal with disasters [19, 20]. Despite the importance of the timely provision of health services, studies indicate that hospitals are poorly prepared for effective disaster response [21–23], which is one of the World Health Organization’s (WHO) primary concerns [7, 24].

Preparedness refers to a set of pre-disaster measures that guarantee an efficient and effective response to a disaster [25, 26]. Preparedness is influenced by various factors that may differ in societies according to their socioeconomic context. East Azerbaijan province is one of the most populated provinces of Iran (more than 4,000,000 people), which is located in the northwest of Iran. This province is one of the disaster-prone areas, and sometimes a part of the province is affected by natural and manmade hazards. The most recent of which are the flash floods of 2017 in Azarshahr and Ajabshir and the twin earthquakes of Arasbaran in 2011. The capital of the province, the city of Tabriz, is located on a fault and has experienced several catastrophic earthquakes throughout history. In this province, 43 hospitals provide medical services.

Hospital preparedness is crucial, especially in areas that are vulnerable to disasters. However, researchers found no previous studies that explored what factors influence the operational preparedness of hospitals in East Azerbaijan, a disaster-prone province. Thus, this study seeks to explore the factors that impact the functional preparedness of hospitals in Tabriz University of Medical Sciences Hospital, a lower middle-income country that faces frequent disasters.

## Methods

### Study design

A descriptive qualitative design was used to explore the opinions of local hospital authorities’ regarding the factors that contribute to hospital preparedness in response to disasters. The study was approved by the Ethics Committee of Tabriz University of Medical Sciences (ID number: IR.TBZMED.REC.1398.133). The authors confirm that all methods were performed according to the relevant guidelines and regulations.

### Study participants

The study participants were disaster management committee members, managers of Tabriz University of Medical Sciences hospitals (17 participants from 12 Hospitals) and experts of East Azerbaijan emergency medical services who are in charge of the provincial hospital’s preparedness who were purposively selected [27]. According to the researchers, they were the most informed people in this field. Detailed characteristics of study participants are presented in Table 1.

**Table 1:** Detailed demographic characteristics and professional background of the study participants

Participants Job title	Number	Age (years)					Gender		Job Experience (year)				Education		
		35–39	40–44	45–49	50–54	55–59	Male	Female	5–10	11–15	16–20	More than 20	Bachelor	Master	Ph.D.
Hospital Manager	8		3	2	1	2	8	0			2	6	2	4	2
Hospital^’^s accreditation officers	1		1				1	0			1		1		
Nursing services heads	1				1		1	0				1		1	
Hospital disaster management officers	3		1	2			3	0				3	3		
Experts of East Azerbaijan emergency medical services	4	1		1		2	4	0			1	3	1	2	1
Total	17	1	5	5	2	4	17	0	0	0	4	13	7	7	3

Including criteria of study participants:

- Being member of hospital disaster management committees with at least five years’ experience.
- Having at least five years of experience in hospital and disaster management.

### Data collection

For data collection, an interview guide was designed and presented to two experts in disaster management. Some revisions were made based on their comments. Two preliminary interviews were conducted to test the interview guide. The interview guide included general questions that were asked at the beginning of the interviews. Based on the interviewee’s answers, more detailed questions were asked to clarify the issue.

After obtaining permission from hospital authorities, Participants were invited by phone to participate in the interview. Two members of the research team (B.S and S.A.A) conducted Semi-structured in-depth interviews in a suitable environment at the participant’s workplaces. Interviews were conducted in Turkish (by B.S.), the native language of study participants, and subsequently translated into English by researchers. Initially, J.B. and B.S. translated the interviews into English, and then J.S. T revised and validated the translations. Each session lasted between 30 and 60 min. Participants^,^ statements were recorded using a tape recorder with their permission. Researchers documented the details of the discussions by taking notes. Researchers transcribed the recordings in Microsoft Word after listening to the recorded voices immediately after each session. After conducting 16 interviews, the researchers felt that the data were saturated because there were no new findings. Another interview was conducted, however, to ensure greater certainty.

### Data analysis

Conventional content analysis was used to explore the data. Two research team members (B.S. and J.B.) separately coded the data manually. Any disagreement between the two researchers regarding data coding was resolved through consensus. If necessary, it was referred to the third researcher (J.S.T.), who had more extensive experience. The steps of data analysis and coding are as follows: Familiarity with the data by repeated listening to the interviewees, getting immersed in data by repeatedly studying the codes, identifying and extracting the primary codes, merging similar codes, putting similar codes together, identifying subcategories, reviewing and concluding explored themes, naming and defining categories, recoding and renaming some categories, and ensuring the reliability of the codes.

### Rigor and accuracy of the findings

Four criteria proposed by Guba and Lincoln [28] were used to improve the rigor and accuracy of the results.

#### Credibility and confirmability

Colleagues’ long-term involvement and experts’ opinions were used for this case. Respondent validity was also used. Researchers summarized the participants’ statements to correct and resolve the wrong and vague cases after the interviews.

#### Dependability

two researchers coded the data to fulfill this criterion.

#### Transferability

the opinions of experts and purposive sampling were used in this regard. In addition to the cases mentioned in this study, the integration methods in the researcher and *transferability* were used.

### Ethics

To meet the ethical research standards, participants received the explanations needed regarding the project’s objectives and methods. Participants’ written consent was obtained. At all stages of the research, the confidentiality principle was observed. Participants were also informed of their right to withdraw from the study at any time.

## Results

**Fig. 1:**
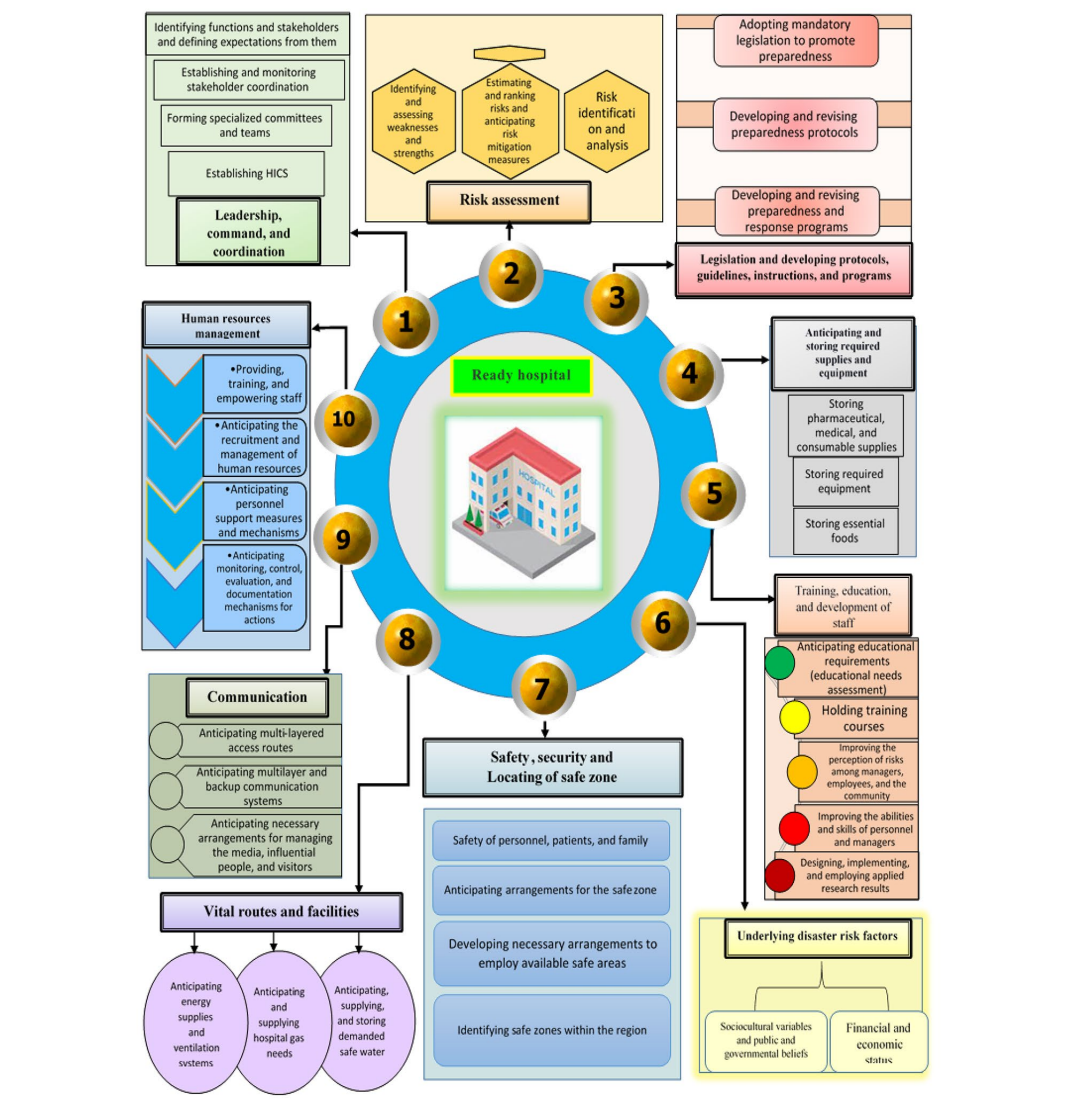
The classification of extracted main and subcategories

Totally 17 interviews were done. Detailed characteristics of study participants are presented in Table 1. The coding process of the interviews resulted in 138 codes (Table 2), which were classified into 10 main categories and 34 subcategories by integrating similar factors (Fig. 1).

**Table 2:** Details of interviews coding and extraction of factors influencing hospitals’ functional preparedness for disasters

Categories	Subcategories	Examples (codes)
**Leadership, command, and coordination**	Identifying functions and stakeholders and defining expectations from them	Identifying and documenting the general and specialized functions/tasks and associated processes (Intra-Sectoral and Inter-Sectoral)
		Identifying stakeholders concerning general, specific, and specialized functions/tasks
		Determining the roles of stakeholders concerning the functions
	Establishing and monitoring stakeholder coordination	Organizing coordination meetings with internal and external stakeholders
		Cooperation contract agreements and developing joint programs with stakeholders
		Constant monitoring of the execution of agreements and programs, as well as comparison with what is anticipated
		Reviewing memoranda and plans and modifying tasks and functions over time
	Forming specialized committees and teams	Coordination with high- and low-ranking (vertical) officials and units, as well as those of the same rank (horizontal)
		The senior administrator of the hospital’s membership in the city’s disaster management headquarters
		Establishing a hospital disaster management committee, convening committee meetings timely, and follow-up approvals
		Forming rapid response teams and DMAT
	Establishing HICS	Launching the hospital incident command system and defining its activation conditions
		Determining the disaster command system members and educating, training, and empowering them.
		Establishing an incident command center in the hospital (Hospital Incident Command Center)
		Anticipating coordination mechanisms in the command hierarchy
**Risk assessment**	Risk identification and analysis	Identifying the hazards to the hospital and the community served
		Analyzing and rating hazards to the hospital and the community served
		Identifying at-risk areas
	Identifying and assessing weaknesses and strengths	Identifying structural and non-structural vulnerabilities of the hospital
		Analyzing and prioritizing vulnerabilities
		identifying hospital facilities and capacities, hospital stakeholders, and hospital partners
	Estimating and ranking risks and anticipating risk mitigation measures	Calculating departmental risks and determining the hospital’s safety index
		Anticipating and implementing risk mitigation measures
		Periodically updating risk assessments and analyzing hazards.
		Documenting and reporting hazards, vulnerabilities, and capacities of the hospital
**Legislation and developing protocols, guidelines, instructions, and programs**	Adopting mandatory legislation to promote preparedness	Requiring all stakeholders to collaborate and coordinate with hospitals to enhance preparedness and response
		Requiring hospitals to improve preparedness through legislation mandating
		Requiring hospitals to use the experiences, guidelines, and recommendations of the World Health Organization and prosperous countries
		The importance of establishing, maintaining, and enhancing hospital preparedness in accreditation metrics
		Increasing the impact of preparedness-related measures in hospital accreditation
		Requiring hospitals to provide preparedness programs to disaster management headquarters of a city / provincial and university (strengthening the demand for hospital upgrade programs)
	Developing and revising preparedness protocols	Developing protocols and guidelines for enhancing hospital preparedness
		Developing and revising protocols and guidelines for developing hospital preparedness programs
		Developing and revising protocols and guidelines for designing response and recovery plans for hospitals
	Developing and revising preparedness and response programs	Developing and revising hospital preparedness and response programs
		Developing and revising SOPs
		Developing, revising, and issuing department and individual job descriptions
**Anticipating and storing required supplies and equipment**	Storing pharmaceutical, medical, and consumable supplies	Preparing a list of pharmaceuticals, medical supplies, and consumables
		Anticipating pharmaceutical needs and storing medical supplies
		Providing the spaces and tools necessary for the proper maintenance of supplies and medications
		Reviewing the expiration dates of stored drugs and supplies and replacing them with new supplies
		Identifying vendors and suppliers of pharmaceuticals and supplies and concluding a memorandum of understanding with them
		Identifying possible centers with similar supplies and drugs and signing a cooperation agreement with them
	Storing required equipment	Anticipating and providing the technical equipment necessary to provide medical services and support
		Anticipating and supplying the emergency equipment required to enhance the capacity
		Periodic equipment inspection, repair, maintenance, and operation assurance
		Preparing medical equipment booklet
		Constant training of personnel operating the equipment
		Identifying suppliers, industries, equipment maintainers, and cooperation contract agreements.
		Identifying possible centers with similar equipment and facilities and signing cooperation agreements with them
	Storing essential foods	Preparing a list of food items needed for emergencies
		Anticipating and providing the food needed in emergencies
		Proper storage and evaluation of their shelf-life
		Replacing consumed and spoiled foods
		Providing proper food storage facilities and equipment
		Identifying food vendors and suppliers and agreeing with them
		Identifying possible centers with food preparation facilities and signing an agreement with them
**Human resources management**	Providing, training, and empowering staff	Assessing the necessary human resources and anticipating how to acquire them
		Anticipating and making arrangements for human resource development.
		Training current personnel and volunteers
	Anticipating the recruitment and management of human resources	Preparing a list of current and potential personnel with their capabilities
		Updating the list of current and potential personnel
		Updating personnel contact information and anticipating replacements
		Anticipating and practicing recruitment techniques and, if necessary, employing personnel
		Preparing job descriptions for individuals, issuing them, and re-issuing them as necessary
		Experience-based appointment of hospital administrators and officials
	Anticipating personnel support measures and mechanisms	Motivating employees and considering monetary and non-monetary incentives for them
		Anticipating and providing essential supplies, food, shelter, and security for employees and their families
		Providing affected personnel with psychological and social services
		Anticipating legal and social protection mechanisms for affected personnel
		Considering reporting and debriefing sessions for after-disasters
	Anticipating monitoring, control, evaluation, and documentation mechanisms for actions	anticipating the recording of actions and experiences
		Anticipating checklists, forms, and methods of performance monitoring and evaluation
		Developing a program for performance monitoring and evaluation
		Performing regular visits
		Anticipating the mechanism of preparing and documenting performance reports
		Identifying those who have learned lessons and anticipating the mechanism of using them
		Providing stakeholders with feedback, evaluations, and recommendations to address deficiencies
Training, education, and development of staff	Anticipating educational requirements (educational needs assessment)	Preparing a required educational needs list (educational needs assessment) for various personnel categories
		Preparing a list of the educational requirements of patients, companions, and the general public
		Making a list of external stakeholders’ educational requirements
		Developing educational curricula and topics based on educational needs priorities
		Developing a timetable for training
	Holding training courses	Implementing training programs at the beginning and throughout the service for various personnel categories
		Educating patients and their families after hospital admission
		Holding university courses related to the disaster
		Evaluating and revising the efficiency of adopted educational programs.
	Improving the perception of risks among managers, employees, and the community	Mandatory courses on disaster management and risk reduction for hospital-related professions.
		Public and general education
		Improve the knowledge and attitudes of managers and stakeholders.
	Improving the abilities and skills of personnel and managers	Designing and carrying out round table, functional, and full-fledged exercises
		Designing and implementing exercises with the participation of all stakeholders
		Practical performance of general and specific functions by all personnel categories
		Evaluating the effectiveness of the exercises and, if necessary, revising them
	Designing, implementing, and employing applied research results	Identifying weaknesses and improvements in preparedness programs
		Developing and conducting applied research
		Constantly reporting research and study results to hospital administrators.
**Vital supplying and facilities**	Anticipating energy supplies and ventilation systems	The proper power supply system and anticipating and providing support systems
		Proper fuel system planning and backup fuel supply systems
		Assessment, supply, and storage of alternative fuels
		Reliable heating and cooling system and support systems
		Educating relevant personnel on the use of storage systems
	Anticipating and supplying hospital gas needs	Anticipating hospital support systems and sustainable gas supply
		Anticipating hospital gases and ensuring their secure storage
	Anticipating, supplying, and storing demanded safe water	Proper water supply and support systems
		Anticipating and storing emergency water supplies
**Communication**	Anticipating multi-layered access routes	Anticipating and providing the helicopter landing site
		Anticipating multiple and separate access routes
		Planning restrictions on hospital entrances and exits
		Preparing maps and geographical information
	Anticipating multilayer and backup communication systems	Improving current communication equipment
		Providing wireless and satellite communication system
		Educating relevant personnel on the use of storage systems
		Designing warning systems for hospitals (such as an alarm)
		Providing and using proper uniforms during times of disaster
	Anticipating necessary arrangements for managing the media, influential people, and visitors	Developing a mechanism to manage visitors
		Managing family and companions of the wounded
		Anticipating how to interact with journalists and media personnel
**Safety and Security** **(Locating and anticipating safe areas)**	Safety of personnel, patients, and family	Taking safety, security, and disciplinary measures
		Managing and avoiding an overabundance of patients
	Anticipating arrangements for the safe zone	Identifying the hospital’s safe zone
		Avoiding the presence of violators and opportunists
		Controlling clients at the hospital’s front entrance
	Developing necessary arrangements to employ available safe areas	Identifying and managing hospital’s safe zones
		Anticipating hospital areas where their function can be altered
		Providing infrastructure for possible safe areas
	Identifying safe zones within the region	Identifying safe zones at the municipal/district level
		Concluding cooperation agreements to provide safe zones
		Updating locations in the region
**Underlying disaster risk factors**	Financial and economic status	The financial situation of the government (such as inflation) and the community
		The hospital’s financial status and the timely delivery of required budgets
		Status of emergency finance
	Sociocultural variables and public and governmental beliefs	General attitudes and beliefs regarding disasters
		The state of public and official perceptions of disaster risk (attitude and belief of managers and people)
		Nationwide public support for program implementation and risk management
		How high-rank organizations support preparedness programs
		The precedence of law and regulation over discrimination and favoritism
		The priority of risk perception programs among the people and the authorities

### Leadership, command, and coordination

Timely and effective response to disasters is needed to close coordination between hospitals’ internal wards and also external stakeholders. The Incident command system is useful for coordination through a unified commanding hierarchy. According to the participants, the first category of preparedness influential factors consists of leadership, command, and coordination. This category has four subcategories: 1-identifying functions, stakeholders, and developing expectations; 2- establishing and monitoring coordination with the stakeholders; 3- forming specialized teams; and 4- establishing Hospital Incident Command System (HICS).

*One of the participants said, “The relevant organizations should be coordinated in advance”; the other stated: “The incident command center must be prepared and equipped.”*

### Risk assessment

Identifying and analyzing hazards, assessing vulnerabilities and capabilities, calculating and prioritizing risks, and anticipating risk reduction interventions were three subcategories in this class. The most significant ideas expressed by participants were:

*“We must evaluate the threats to our hospitals. Evaluate our vulnerability. Assess our capability. Specify the risk type. Then we can assess and estimate the extent of the damage. Estimate the severity and occurrence and recurrence probabilities.“, “We need a comprehensive risk assessment.”*

### Legislating and developing protocols, guidelines, and programs

Developing and implementing preparedness programs should be supported by legal mechanisms. The participants emphasized it, and there were many codes in this category that were classified into 3 subcategories: enacting laws mandating preparedness, developing and updating preparation protocols, and developing and updating preparedness and response programs.

*“Disasters have a set of metrics-based guidelines outlined in the accreditation program.“, “We have two points: one, the hospital’s legal obligations, and second, the manager’s attitude on preparedness.”*

### Estimating and storing supplies and equipment

Estimating and storing standard levels of supplies and equipment were essentials that most experts agreed on providing them on schedule. The three subcategories extracted in this category were: storage of medicines and medical supplies, storage of essential equipment, and storage of essential foods.

*“We must constantly prepare and store certain medications, foods, and consumables and replace them according to their expiration dates.“, “Support should be considered for a variety of facilities and equipment.”*

### Human resources management

According to some interviewees, human resource management was one of the important factors in making the best use of existing capacities and providing effective disaster response. This class was comprised of four subcategories, and the participants expressed:

*“Human resources and how they are organized are factors that may contribute to the successful and optimum management of the disaster.“, “In times of disaster, staffing shortages, especially in law enforcement, security, and services, are one of our most prevalent issues*.”

### Education, training, and development of staff

Nearly all participants mentioned that staff education, training, and development are the most crucial factors. This category includes anticipating educational needs (educational needs assessment), conducting academic courses, enhancing the perception of managers, staff, and the community, improving the capability and skills of staff and managers, and designing, implementing, and utilizing the results of applied research.

*“Through the training, the staff knows how to act.“, “Human resource and healthcare staff training is highly effective for handling all types of crises.“, “In addition to training, maneuvers must be carried out.“, “Organizations involved in the disaster, such as the fire department and the Red Crescent, should also be invited to participate in joint exercises and maneuvers.”*

### Vital routes and facilities

Important routes and facilities have also been identified as significant themes in hospitals’ functional preparedness. Three subcategories of influential factors are raised from analyzing interviews: anticipating energy supplies and ventilation systems; anticipating and supplying hospital gas needs; and anticipating, supplying, and storing demanded safe water.

*“Support facilities, such as the power system, should be determined and documented in advance of any event.“, “… for instance, the hospital’s power supply system. In the event of a power outage, the question of whether there is a suitable replacement and support. To what extent can the hospital’s water resources meet its needs in the event of a water shortage?”*

### Communication

Communication is one of the essential functions that are disrupted at the beginning of disasters. Hospitals’ performance depends on internal and external communications. Thus it was considered by study participants. Three subcategories related to communication were anticipating multilayered access routes, multilayer and backup communication systems, and the necessary arrangements for managing media, famous figures, and visitors.

*“We were required to construct a helipad.“, “During times of disaster, information and communication networks often have difficulties; thus, the required arrangements should be made for backup and alternative systems.”*

### Safety, security, and locating of safe zones

Disasters may destroy hospitals, damage structures, and become unsafe places for patients and workers. The hospitals and patients may need to be evacuated. A large number of injured and their families rush to hospitals. Therefore it is necessary to pay attention to the safety and security of hospitals. The study participants considered these issues vital. They emphasized it, and this category was formed by combining the following four subcategories according to their opinions: safety of staff, patients, and their families; anticipating security plans; making preparations to utilize available safe zones and locating available safe zones in the region.

*“At least at the hospital’s entrance, we should control who and how patients are brought into the hospital. Security concerns should be addressed because, in the meantime, some individuals might exploit the situation.“, “A contract with the police is essential for controlling hospital security.“, “We intended to use the specific area and hospital grounds to group the patients with the tents.“.*

### Underlying disaster risk factors

In the final identified theme, financial and economic status and socio-cultural elements and attitudes of people and officials were two subcategories that included essential determinants of the functional preparedness of hospitals in response to disasters.

The following are some of the topics discussed by the participants:

*“The status of the community also influences many concerns due to inflation and economic challenges.“, “They face challenges with patient congestion, numerous complaints, high expectations, a shortage of equipment, and, most significantly, economic sanctions that impact them all.” “Disaster management, especially preparedness, is costly. The hospital has limited financial resources.”*

## Discussion

The present qualitative research explored the influential factors on the functional preparedness of hospitals in response to disasters; these factors were extracted and categorized into ten main categories- and 34 subcategories based on participants’ opinions.

Evidence shows that for an effective response; functions and related stakeholders should be identified, documented, and prioritized. It is necessary to define collaboration details and establish reciprocal expectations by organizing coordination meetings [29]. Disorganization, parallel execution, egocentric behavior, and wasting resources disrupt the service delivery process [30, 31]. Ellen A. et al. stated that using the hospital incident command system necessitated careful leadership during a disaster and its activation requires special conditions that must be determined by clear internal organizational policies [32]. Developing an incident command system minimizes confusion, chaos, and loss of life and property, accelerates coordination and control in providing effective responses, increases resilience, and optimize using of resources [33–35]. In another study, Samsuddin et al. identified the formation of hospital disaster management committees as one of the influential factors in hospital preparedness and resilience [36]. Regarding specialized disaster management teams, Ahmadi and Manoochehr et al. reported that accurate environmental assessments, teamwork, and effective internal and external communication are crucial for the effectiveness of the response phase [37].

Risk assessment is a significant factor in hospital functional preparedness. According to Seyedin et al., risk assessment of hospitals is an essential factor that improves hospital preparedness by altering managers’ attitudes. Notably, when executed with more outstanding care, the results would be more favorable [30].

According to Chan et al., proper planning is the first step in the preparation cycle [38, 39]. Periodic evaluation and constant updating of protocols, preparedness, and response programs are necessary for enhancing preparedness [40]. The current study participants said that the enactment of mandatory preparedness laws is necessary to facilitate the implementation of the various aspects of preparedness programs. According to the study by Seyedin et al., one of the most significant obstacles to preparedness is the applicability of established laws [30].

Estimation and storage of equipment, medical supplies, medications, and foods, providing vital routes and appropriate facilities, additional energy supply systems, hospital gases, safe water, and optimal use of available areas were other influential explored factors. In this regard, several studies identified proper support as the main precondition for providing constant services, and due to its direct correlation with reducing disaster-related fatalities and complications, studies recommend maximizing support and logistical factors [41–43].

Based on the experts’ view, proper organization and human resources management during disasters are the main concerns of hospitals. The findings of Amerion et al. demonstrate the significance of human resources as one of the foundational pillars of hospital services management and consider improper organization and unnecessary relocation as the causes of human resources waste [44]. These results align with the findings of this study. According to Zaboli et al., employees need to consider their own and their family’s health [45]. As the proper performance of employees depends on their mental and emotional preparedness, the alleviation of their concerns, and the management of their stress, anticipating supportive and motivational mechanisms may be effective in alleviating concerns and improving the performance of employees during the response phase. Training, practicing, and keeping employees up-to-date is one of the most crucial and essential aspects of hospital preparedness. Intriguingly, it is consistent with the present study and highlights the significance of this factor relative to others [46, 47].

Some studies suggest that training in disaster management improves individuals’ knowledge and skills [18, 48]. Following the investigations of Los Angeles hospitals, Kaji et al. found that hospitals cannot be functionally prepared without coherent training programs and courses. These results were obtained when the hospitals have a high level of equipment and facilities [49]. Also, Irannejad et al. emphasized theoretical and practical training for all levels [50].

In the initial minutes following a disaster, anticipating access routes, multi-layered information, and communication systems, planning for managing the media, key individuals, and visitors effectively reduces disorder, confusion, lack of coordination, and the number of initial casualties and damages. Aitken and Leggat et al. considered the unreliability, inefficiency, and inadequacy of relying solely on one communication method in emergencies as a root cause of other potential crises [51]. Moreover, in an investigation of Japanese hospitals, Mulyasari et al. concluded that using alternative and backup communication devices is an important indicator of preparedness [52].

Experience has shown that effective implementation of preparedness programs and appropriate response operations cannot occur without ensuring the safety of personnel, patients, and others and taking physical and equipment security precautions. In this regard, the study by Edbert et al. suggested that hospital personnel should have the necessary familiarity with security issues through continuous training, and their knowledge and awareness regarding security laws and instructions must be monitored and assessed [53]. Several other studies have reported that the lack of security programs is the reason for the poor preparedness of hospitals in disasters [54, 55].

It is undeniable that financial and economic status, sociocultural factors, and the beliefs of people and officials all contribute to the functional readiness of hospitals. In this regard, J Cliff B. et al. reported, based on a study of hospitals in the United States, that even assuming the availability of all facilities and resources, the optimal level of preparation will not be attained without a proper understanding of the risks [56].

### Limitations

In this study, the private and charitable hospitals were not included. Because in East Azerbaijan province, these hospitals have a different structure, the opinions of their managers may be different about the factors influencing the preparedness of hospitals.

## Conclusion

Hospitals’ preparedness for an effective response to disasters is a vital issue that requires the attention of all stakeholders. This study identified 10 categories of factors that influence the preparedness of hospitals in response to disasters. These factors should be considered systematically by the hospital managers. By addressing these factors, it can be expected that hospitals’ preparedness will be improved.

### Electronic supplementary material

Below is the link to the electronic supplementary material.

Supplementary Material 1

## Data Availability

All data generated or analyzed during this study are included in this published article.

## References

[1] Bani Hashemi SASF (2012). Assessment of climate change and its effects in Iran. Jungles and Grasslands.

[2] Jahangiri K, TABIBI S. Disaster management: designing a new model for effective planning in bioterrorism. 2003.

[3] Torani S, Majd PM, Maroufi SS, Dowlati M, Sheikhi RA. The importance of education on disasters and emergencies: a review article. J Educ Health Promotion 2019, 8. 10.4103/jehp.jehp_262_18 PMC6512217 31143802

[4] Yarmohammadian M, Nasr Isfahani M, Anbari E (2016). Assessment of preparedness and response of teaching hospitals of Isfahan, Iran, to chemical, biological, radiological, and nuclear incidents. Health Inf Manag.

[5] CRED.: 2021 Disasters in Numbers. In. Brussels; 2022.

[6] Nivolianitou Z, Synodinou B (2011). Towards emergency management of natural disasters and critical accidents: the Greek experience. J Environ Manage.

[7] Heidaranlu E, Ebadi A, Khankeh HR, Ardalan A. Hospital disaster preparedness tools: a systematic review. PLoS Curr 2015, 7. 10.1371/currents.dis.7a1ab3c89e4b433292851e349533fd77 PMC4575155 26425401

[8] Green GB, Modi S, Lunney K, Thomas TL (2003). Generic evaluation methods for disaster drills in developing countries. Ann Emerg Med.

[9] Khankeh HR, Khorasani-Zavareh D, Johanson E, Mohammadi R, Ahmadi F, Mohammadi R (2011). Disaster health-related challenges and requirements: a grounded theory study in Iran. Prehosp Disaster Med.

[10] Ahmadi A, Bazargan-Hejazi S (2018). 2017 Kermanshah earthquake; lessons learned. J Injury Violence Res.

[11] Pouraghaei M, Jannati A, Moharamzadeh P, Ghaffarzad A, Far MH, Babaie J (2017). Challenges of hospital response to the twin earthquakes of August 21, 2012, in East Azerbaijan, Iran. Disaster Med Pub Health Prep.

[12] Amiri M, Mohammadi G, Khosravi A, Chaman R, Arabi M, Sadeghi E, Kalatejari M. Hospital preparedness of Semnan province to deal with disasters. 2011.

[13] Mohebbifar R, Tabibi S, Asefzadeh S (2008). Designing a structure of disaster management for Iran. J Health Adm.

[14] Ardalan AKM, Osooli M, Shamseddini A, Zare M, Moosavand AK, Mirsanei R, Samadi S. Profile of Natural hazards in I.R.Iran. In. & Emergency Health Academy. Iran’s National Institute of Health Research and SPH of Tehran University of Medical Sciences; 2012. Disaster.

[15] Nafar H, Tahmazi Aghdam E, Derakhshani N, Sani’ee N, Sharifian S, Goharinezhad S (2021). A systematic mapping review of factors associated with willingness to work under emergency conditions. Hum Resour Health.

[16] Derakhshani N, Maleki M, Pourasghari H, Azami-Aghdash S (2021). The influential factors for achieving universal health coverage in Iran: a multimethod study. BMC Health Serv Res.

[17] Djalali A, Castren M, Hosseinijenab V, Khatib M, Ohlen G, Kurland L (2012). Hospital Incident Command System (HICS) performance in Iran; decision making during disasters. Scand J Trauma Resusc Emerg Med.

[18] Djalali A, Hosseinijenab V, Hasani A, Shirmardi K, Castrén M, Öhlén G, Panahi F. A fundamental, national, medical disaster management plan: an education-based model. 2012. 10.1017/s1049023x00007524 20301078

[19] Naser WN, Ingrassia PL, Aladhrae S, Abdulraheem WA (2018). A study of hospital disaster preparedness in South Yemen. Prehosp Disaster Med.

[20] Jahangiri K, Izadkhah YO, Lari A (2014). Hospital safety index (HSI) analysis in confronting disasters: a case study from Iran. Int J Health Syst Disaster Manage.

[21] Ghanbari V, Maddah S, Khankeh H, Karimloo M (2011). The effect of a disaster nursing education program on nurses’ preparedness for responding to probable natural disasters. Iran J Nurs.

[22] Khankeh H. Designing a Comprehensive Model for Health Disaster Management. Ph. D Dissertation [in Persian]; 2007.

[23] Shokouh SMH, Anjomshoa M, Mousavi SM, Sadeghifar J, Armoun B, Rezapour A, Arab M (2014). Prerequisites of preparedness against earthquake in hospital system: a survey from Iran. Global J Health Sci.

[24] Guha-Sapir DHP, Below R. Annual Disaster Statistical Review 2015: The numbers and trends. 2016.

[25] Nations U. UNISDR terminology on disaster risk reduction. *United Nations Office for Disaster Risk Reduction, Report* 2009.

[26] Liguori N, Tarque N, Bambaren C, Santa-Cruz S, Palomino J, Laterza M (2019). Basic seismic response capability of hospitals in Lima, Peru. Disaster Med Pub Health Prep.

[27] Palinkas LA, Horwitz SM, Green CA, Wisdom JP, Duan N, Hoagwood K (2015). Purposeful sampling for qualitative data collection and analysis in mixed method implementation research. Adm Policy Mental Health Mental Health Serv Res.

[28] Lincoln YS, Guba EG. Naturalistic inquiry: sage; 1985.

[29] Management MHD. Disaster Management Model for the Health Sector: Guideline for Program Development. In.: Government of Manitoba Manitoba, Canada; 2002.

[30] Seyedin H, Moradimajd P, Bagheri H, Nasiri A (2021). Providing a chemical events and threat’s preparedness model for hospitals in the country: a qualitative study. J Mil Med.

[31] Khankeh HR, Mohammadi R, Ahmadi F, Maddah SSB, Ranjbar M, Khodaei MR (2006). Management of Health Care services at Time of Natural disasters. Archives of Rehabilitation.

[32] Love EA, Degen SC, Craig JE, Helmers RA (2021). Activating the Hospital Incident Command System response in a Community Specialty Practice: the Mayo Clinic Experience. WMJ.

[33] Shooshtari S, Tofighi S, Abbasi S. Benefits, barriers, and limitations on the use of Hospital Incident Command System. J Res Med Sciences: Official J Isfahan Univ Med Sci 2017, 22. 10.4103/1735-1995.202146 PMC5393096 28465695

[34] Farcas A, Ko J, Chan J, Malik S, Nono L, Chiampas G (2021). Use of incident command system for disaster preparedness: a model for an emergency department COVID-19 response. Disaster Med Pub Health Prep.

[35] Jagminas L, Bubly G (2003). The hospital emergency incident command system-are you ready?. Rhode Island Medical Journal.

[36] Samsuddin NM, Takim R, Nawawi AH, Alwee SNAS (2018). Disaster preparedness attributes and hospital’s resilience in Malaysia. Procedia Eng.

[37] Ahmadi A, Manoochehri S (2020). Assessing the status and analysis of factors affecting the desirability of Crisis Management of Environmental hazards in Ghaenat City. Spat Plann.

[38] JTS Chan RY, Tang SYH (2002). Hospital preparedness for chemical and biological incidents in Hong Kong. Hong Kong Med J.

[39] Boen T, Gibbs T (2009). Safe in an emergency. World Health Organization Bulletin of the World Health Organization.

[40] Etchegaray JM, Thomas EJ (2012). Comparing two safety culture surveys: safety attitudes questionnaire and hospital survey on patient safety. BMJ Qual Saf.

[41] May G (2013). Disaster readiness for ES departments. Health Facil Manag.

[42] Supe A, Satoskar R (2008). Health services responses to disasters in Mumbai sharing experiences. Indian J Med Sci.

[43] Vahedparast H, Ravanipour M, Hajinezhad F, Kamali F, Gharibi T, Bagherzadeh R (2013). Assessing hospital disaster preparedness of Bushehr province. ISMJ.

[44] Amerion A, Delaavari A, Teymourzadeh E (2010). Rate of preparedness in confronting crisis in three selected border hospitals. J Mil Med.

[45] Zaboli R, TOUFIGHI S, Amerioun A, Moghadasi H. Survey of Tehran city hospitals preparedness for disaster. 2006.

[46] Veenema TG (2006). Expanding educational opportunities in disaster response and emergency preparedness for nurses. Nurs Educ Perspect.

[47] Zaboli R, Sh T, Seyyedin S, Malmoon Z, Hoseini Shokuh S (2009). Organizational vulnerability and management of clinical departments against crisis. J Mil Med.

[48] Collander B, Green B, Millo Y, Shamloo C, Donnellan J, DeAtley C (2008). Development of an all-hazards hospital disaster preparedness training course utilizing multi-modality teaching. Prehosp Disaster Med.

[49] Kaji AH, Lewis RJ (2006). Hospital disaster preparedness in Los Angeles county. Acad Emerg Med.

[50] Irannejad B, Safarabadi M, Jadidi A (2017). Survey of biological incidents preparedness of hospitals in Markazi Province in 2016. J Mil Med.

[51] Aitken P, Leggat P. Considerations in mass casualty and disaster management. Emerg Medicine: Int Perspective 2012:145–82.

[52] Mulyasari F, Inoue S, Prashar S, Isayama K, Basu M, Srivastava N, Shaw R (2013). Disaster preparedness: looking through the lens of hospitals in Japan. Int J Disaster Risk Sci.

[53] Hsu EB, Thomas TL, Bass EB, Whyne D, Kelen GD, Green GB (2006). Healthcare worker competencies for disaster training. BMC Med Educ.

[54] Wang T-L, Chen H-T, Chang H. Hospital preparedness for weapons of mass destruction incidents: an initial assessment. Ann Disaster Med Vol 2004, 2(2).

[55] Maleki M, Shojaie P (2007). Hospitals preparation in disasters: security. J Health Adm.

[56] Cliff BJ, Morlock L, Curtis AB (2009). Is there an association between risk perception and disaster preparedness in rural US hospitals?. Prehosp Disaster Med.

