# Supplementary material for: Exploring hospitals’ functional preparedness effective factors in response to disasters: a qualitative study in a lower middle-income country
Source: BMC Health Serv Res. 2024 Feb 13;24:197. doi: 10.1186/s12913-024-10630-y (PMC10865653; doi:10.1186/s12913-024-10630-y)
Supplement: Supplementary file 1 — Supplementary Material 1 [file 12913_2024_10630_MOESM1_ESM.docx]

**Interviews guide:**

1. In your opinion, what factors make some hospitals pay attention to disaster preparedness?
2. In your opinion, what measures should be taken to improve the hospital's functional preparedness against disasters?
3. In your opinion, what factors influence the hospital's functional preparedness against disasters?
4. To what extent do you estimate the role of each of the mentioned influential factors in the hospital's functional preparedness?
